# Controlling systems and controlling legacies: Barbara McClintock’s 1961 conversation with two bacterial geneticists

**DOI:** 10.1007/s40656-024-00631-9

**Published:** 2024-09-12

**Authors:** Qinyan Wu

**Affiliations:** History and Sociology of Science Department, University of Pennsylvania, Claudia Cohen Hall, 249 S. 36th Street, Philadelphia, PA 19104 USA

**Keywords:** Barbara McClintock, François Jacob, Jacques Monod, The Nobel Prize, The Matilda Effect, Gender-neutral Science

## Abstract

Barbara McClintock (1902–1992), the renowned American maize geneticist, received the 1983 Nobel Prize “for her discovery of mobile genetic elements,” becoming the seventh woman scientist to receive a Nobel Prize. However, Nathaniel Comfort points out that McClintock viewed her primary contribution as the elucidation of control systems, rather than the discovery of mobile elements. McClintock’s interest in control systems dates back to the 1940s, and this paper investigates her 1961 conversation with François Jacob and Jacques Monod, where she sought to shape the interpretation of her work by drawing parallels between maize control systems and a bacterial system they had recently discovered. Despite McClintock’s efforts, Jacob and Monod rejected her parallels and suggested that her contribution was limited to mobile elements. Through an examination of their published papers, I argue that Jacob and Monod’s rejection stemmed from their failure to fully comprehend maize control systems. Disciplinary discrepancy helps explain Jacob and Monod’s lack of comprehension: they were molecular geneticists working on bacteria, while McClintock was a classical geneticist studying maize. I further argue that gender played a role, as McClintock experienced the Matilda effect—the under-recognition of her contribution, reinforced by the reactions of two male geneticists, and ironically, by the award of the Nobel Prize. Control systems, stemming from McClintock’s reverence for organisms, embodied what Evelyn Fox Keller defines as “gender-neutral science.” This divergent view of science provides insight into why Jacob and Monod failed to grasp McClintock’s work in 1961.

## Introduction: Mobility? Control!

When Barbara McClintock received the Nobel Prize at the age of 81 in 1983, news reports flooded in to celebrate the first woman scientist’s unshared prize in Physiology and Medicine. She was awarded “for her discovery of mobile genetic elements,”^1^ vividly known as the jumping genes. This acclaim suggests that McClintock’s major contribution was mobile elements, also named transposable elements. In Evelyn Fox Keller’s influential biography, *A Feeling for the Organism* (1983a), mobile elements centered McClintock’s career—initially underappreciated in the late 1940s and 1950s, they were rediscovered and credited in the 1970s. McClintock’s enduring legacy includes the publication of a paper collection, *The Discovery and Characterization of Transposable Elements* (1987), and a recollection from her colleagues, *The Dynamic Genome* (1992), featuring a substantial section on “Transposition.” With mobility the central theme, McClintock’s story was fulfilled with the long-deserved Nobel Prize.

After McClintock’s passing in 1992, and with the availability of her correspondences and research notes, Nathaniel Comfort questions Keller’s narrative, highlighting “the fact that McClintock did not win the prize for what she thought was most important in her work” (2001a, p. 475). To Comfort, it is a *fact* that McClintock focused not on mobility, but on control—the controlling effects of controlling elements in maize control systems. Comfort (1999) argues that geneticists in the late 1940s already accepted mobile elements, but failed to appreciate their controlling effects. The Nobel Prize, therefore, only perpetuated the myth that mobility centered McClintock’s research. During the ceremony, McClintock delivered a lecture titled “The Significance of Responses of the Genome to Challenge,” stating: “However, I am very much interested in the *nature* of *changes* that occur in the genome, when the genome meets something very unexpected.”^2^ Mobility was not her keyword. In her lecture’s written form, McClintock restated: “The mobility of these activated elements allows them to enter different gene loci and to take over control of action of the gene wherever one may enter” (1987, p. 634). The elements transpose to control genetic action, so the genome responds to environmental changes. Therefore, while McClintock did refer to mobile elements, as Comfort points out, mobile elements are not the whole picture; the real significance lies in control.

The Nobel lecture was not the sole occasion on which McClintock asserted her focus on control rather than mobility. A pivotal moment occurred in 1961, when she proposed parallels between maize control systems and a bacterial system discovered by two bacterial geneticists, François Jacob and Jacques Monod. However, Jacob and Monod declined the parallels and emphasized that McClintock’s contribution lay in mobile elements. Nobel Prizes sided with Jacob and Monod—they immediately received it in 1965 for the bacterial system, later known as the operon, while McClintock received the 1983 prize for mobile elements. A reevaluation of this historical moment reveals that McClintock had valid reasons to draw the parallels, but Jacob and Monod failed to grasp maize control systems. Correspondence and lecture manuscripts showcase McClintock’s enduring interest in the parallels between maize and bacteria, though she grew critical of geneticists’ overconfidence in operon’s universality in the 1970s.

McClintock’s legacy transcends her scientific achievements. She provided long-term inspiration for Evelyn Fox Keller, who interprets mobile elements as gender-neutral science based on a feeling for the organism. If control, instead of mobility, centered McClintock’s research, should gender still matter? Nathaniel Comfort suggests: “[the] reception of controlling elements is a poor model for gendered discrimination in science” (1999, p. 153). However, I argue that the rapid dismissal of her argument by two prominent male geneticists resonates with many other cases of gendered bias at the time, as documented in Margaret Rossiter (1995)’s influential account of women scientists in the twentieth century. Maize control systems stem from McClintock’s reverence for organisms, which differs from Jacob’s and Monod’s views. Keller’s concept of gender-neutral science helps explain why McClintock understood Jacob and Monod’s research but not vice versa. The 1961 conversation serves as a reminder of the multifaceted role gender plays in science—even prominent women scientists like McClintock faced under-recognition of their work.

## Technical details: 3 control systems + 1 mobile element

Nathaniel Comfort highlights the disconnection between the 1983 Nobel prize and McClintock’s primary research on control systems, suggesting: “the reader may wonder whether she was really right or wrong, whether she deserved the accolades she received” (2001b, p. 15). In assessing the merit of control systems, I scrutinize McClintock’s argument of drawing parallels between her research and a bacterial system discovered by François Jacob and Jacques Monod, who received the 1965 Nobel prize. This section elucidates four key technical terms crucial to understanding the parallels: the *Ac-Ds* system and the *Spm* system in maize; the regulator-operator system and the episome element in Escherichia coli (*E. coli*). Their methodologies also differed: McClintock focused on maize, a higher organism with ten pairs of chromosomes, employing a pre-molecular and cytological approach – she bred and crossed maize of certain phenotypes and observed the chromosomes under microscopes. In contrast, Jacob and Monod studied *E. coli*, a prokaryote with a single chromosome ring, taking a molecular and biochemical approach. The disciplinary discrepancy between cytogenetics and molecular biology anticipates Jacob and Monod’s lack of understanding of maize control systems.

McClintock started researching the *Ac-Ds* system in the late 1940s and presented it at the 1951 Cold Spring Harbor Symposium, titled “Chromosome Organization and Genic Expression.” To summarize, *Ac* is a chromosomal factor that activates *Ds*, another chromosomal factor; *Ds* further causes a chromatid, one of the two chromosomal strands, to break. I break down McClintock’s research into 4 steps: First, she noticed a few kernels with special color spots and observed their chromosomes during the pachytene stage of meiosis. Second, she found that chromosome 9 was always partly broken and defined the *Ds* factor–meaning dissociation—as responsible for the breakage. Third, McClintock observed that chromosome 9 did not always break on the same locus, attributing this variability to the transposition of *Ds* within one chromatid or sometimes to other chromosomes. She mapped out a few gene loci where *Ds* had transposed to, caused chromosomal breakage, and led to phenotypical variations. Fourth, McClintock introduced the *Ac* factor which activates *Ds*: “*Ac* is a dominant factor that must be present if any breakage events are to occur at *Ds*” (1987, p. 232). *Ac* can also transpose; as long as *Ac* is present in the nucleus, *Ds* can cause breakage. In addition, maize progeny’s phenotypes followed mendelian inheritance during cross- and self-pollination experiments, which validated *Ac* and *Ds* as chromosomal factors. The control chain is *Ac*–*Ds*–other chromosomal segments, resulting in phenotypical variations. Regarding mobility, both *Ds* and *Ac* can transpose.

McClintock proposed the concept of genetic control and distinguished controlling elements, such as *Ac* and *Ds*, from gene elements in her presentation “Controlling Elements and the Gene” at the 1956 Cold Spring Harbor Symposia, which François Jacob attended. During the presentation, she reported another controlling element—the suppressor-mutator element, abbreviated as the *Spm* element.^3^ It is crucial that the suppressor-mutator element is a *single* element that suppresses *or* mutates a chromosomal locus. I summarize McClintock’s discovery into 3 steps: First, McClintock noticed that some kernels were pigmented in anthocyanin, a bluish-purple color. Second, observing the chromosomes, she attributed the pigment to a few modified loci such as a_1_^m−1^ on chromosome 3. She proposed that these loci controlled the expression of nearby segments. Third, McClintock discovered that a_1_^m−1^ was further controlled by a transposable element, the *Spm* element. The *Spm* element could either *suppress* the expression of a_1_^m−1^, leading to light anthocyanin spots on the kernels, or *cause an inheritable mutation* at a_1_^m−1^, resulting in intense anthocyanin blotches. The dual function coined the name “suppressor-mutator” for the *Spm* element. The control chain is *Spm*– a_1_^m−1^–gene elements, namely the *Spm*–a_1_^m−1^ system. In the 1950s, McClintock discovered multiple mutable loci including a_1_^m−5^, a_2_^m−8^, and wx^m−8^, all susceptible to suppression or mutation by a specific *Spm* element. She termed this shared control mechanism as “the suppressor-mutator system,” abbreviated as “the *Spm* system.” Despite its name suggesting only the *Spm* element, the *Spm* system comprises two controlling elements—the *Spm* element and a mutable locus. Notably, only the *Spm* element is mobile.

*Ac-Ds* system and the *Spm* system illustrate two main kinds of controlling effects. McClintock elucidated the relationship between mobility and control at the 1956 symposium: “[controlling] elements were initially discovered because they do not remain at one position in the chromosome complement” – *Ds* initially drew her attention by causing chromosome 9 to break at various locations (1987, p. 345). She continued: “[transposability], which made possible the recognition of controlling elements in the chromosome complement of maize, may not serve in all cases as a reliable criterion for discrimination between [controlling elements and gene elements] because the frequency of its occurrence may be so low, under certain conditions, that detection may be difficult” (p. 363). McClintock regarded mobility as playing a supportive role that should retreat from the spotlight after introducing the controlling effects. Therefore, she termed these elements as controlling elements, rather than transposable elements. She also published findings about controlling elements which do not transpose, such as the subsection titled “control of gene action by a non-transposing *Ds* element” in “Mutation in Maize (1956).”

By prioritizing control, McClintock aimed to grasp the larger picture of organism development. As early as 1951, she proposed two functions of controlling systems: First, they may explain cellular differentiation, i.e., why cells within a higher organism develop into diverse forms from the same hereditary material. Second, they help organisms respond to environmental changes: “It is tempting to consider that changed environmental conditions may well alter otherwise-established rates of reaction, and thus initiate alterations in the nuclear components at predictable times, leading to strikingly modified phenotypic expression” (1987, p. 253). Organisms may adapt to environmental changes by modifying their nuclear components—the *Ac-Ds* system causes chromatids to break and the *Spm* element induces mutations, resulting in phenotypical variations. McClintock’s lifelong interest in linking controlling effects to organisms’ responses to the environment was evident in her Nobel lecture in 1983. To delve deeper into this relationship, she cited other geneticists’ studies on *Drosophila*, a genus of flies, and *Oenothera*, a genus of flowering plants.

No wonder McClintock noticed François Jacob and Jacques Monod’s paper, “Gènes de structure et gènes de regulation dans la biosynthèse des proteins (Genes of Structure and Genes of Regulation in the Biosynthesis of Proteins),” published in Jacob & Monod 1959. She took an immense interest and translated it from French to English on her own.^4^ As indicated by the title, Jacob and Monod posited the two types of genes: genes of structure and genes of regulation, which resonates with McClintock’s classification of gene elements and controlling elements, although they did not cite her work. Under this classification, Jacob and Monod proposed the regulator-operator system in *E. coli*, a single-cell prokaryote with a single chromosome ring. They illustrated the system in a short essay “L’opéron: groupe de gènes à expression coordonnée par un opérateur (Operon: a Group of Genes with the Expression Coordinated by an Operator)” (Jacob et al., 2005), and “Genetic Regulatory Mechanisms in the Synthesis of Proteins” (Jacob & Monod, 1961a).^5^

Based on the three papers, I summarize their research process into 4 steps: First, according to previous studies, *E. coli* synthesize two kinds of proteins to digest lactose: β-galactosidase breaks lactose into two simple sugars, glucose and galactose; galactoside permease transports lactose into the cell. The proteins are encoded by two adjacent genes, y and z, respectively. Jacob and Monod classified *y* and *z* as structural genes. Second, *E. coli* only synthesize the proteins when lactose is available. Jacob and Monod attributed this self-regulation to another gene, *i*^+^, termed the regulator. They noticed *i*^+^ because its mutants failed self-regulation: when *i*^+^ mutated to *i*^*−*^, proteins were produced without lactose; when *i*^+^ mutated to *i*^*s*^, *E. coli* stopped the synthesis even in the presence of lactose. Third, the regulator gene *i* (including *i*^+^,* i*^*−*^, and *i*^*s*^) is not adjacent to *z* and *y*. Jacob and Monod introduced a fourth gene *o*^+^, the operator gene, as an intermediary for the regulator to control the structural genes. When* o*^+^ mutated to *o*^*c*^, *E. coli* synthesized proteins even without lactose. Finally, based on mutant behaviors, Jacob and Monod deduced that in the absence of mutants, the operator gene *o* directs the structural genes, *y* and *z*, to constantly synthesize proteins, while the regulator gene *i* produces a substance, termed as the repressor, to repress *o* and halt synthesis unless lactose is available. In other words, *i*^+^ responds to lactose and *o*^+^ responds to the repressor, while mutants lose sensitivity, disabling the system.

**Fig. 1:**
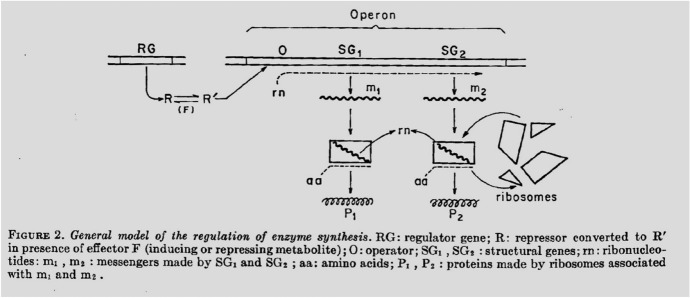
Jacob and Monod’s cartoon-like diagram for the regulator-operator system (1961b, p. 194), used with permission from Cold Spring Harbor Laboratory Press

The regulator and the operator are classified as genes of regulation, responsible for controlling the structural genes to synthesize proteins. The regulator-operator system involves non-genetic elements such as proteins and the repressor. Precisely, the repressor is a cytoplasmic substance generated by the regulator, inhibiting the operator from activating the structural genes. The suppression mechanism serves as the default setting for *E. coli* to digest lactose. Jacob and Monod drew a cartoon-like diagram to visualize the system (Fig. 1).

The diagram portrays the entire system, later known as the operon model, earning the 1965 Nobel Prize in Physiology or Medicine. The genetic components, depicted as two parallel lines, show the control chain: “regulator (RG)–operator (O)–structural genes (SG_1_ and SG_2_).”

Jacob and Monod did not cite McClintock in their papers, though Jacob attended the 1956 Cold Spring Symposia where McClintock proposed to distinguish gene elements from controlling elements. Instead, Jacob likened McClintock’s controlling elements to a mobile element, the episome. In the 1950s, Jacob collaborated with geneticists such as Élie Wollman and studied temperate bacteriophage (shortened as phage), a virus that infects *E. coli*. In his 1958 Harvey lecture titled “Genetic Control of Viral Functions,” Jacob proposed that upon infecting *E. coli*, the phage assumes either the vegetative state, where it replicates independently within the bacterial host, or the prophage state, where it inserts a genetic segment into the bacterial chromosome and replicates with the bacteria. The distinction between the two states depends on the insertion of a genetic segment: “[in] certain circumstances, changes from one state to another may occur. The term *episome*, or *episomic element*, has been proposed to designate this particular genetic element” (Jacob 1960, 1959 pp. 31–32). The episome is defined by its capacity to transpose between different organisms, not restricted solely to phages. For instance, during the mating process between male and female *E. coli*, certain genetic segments known as sexual factors can transpose from the male to the female organism. Jacob identified these sexual factors as episomes due to their mobility. Jacob primarily emphasized episomes’ mobility and only hinted at their impact on the expression of adjacent genes. He cited McClintock, drawing a parallel based on mobility: “In maize the factors described as ‘controlling elements’ (McClintock, 1951) also appear to behave as episomes, since these agents are not always present, but when present they are added to certain chromosomal sites and can move from one site to another, and even from one chromosome to another” (p. 32). While McClintock highlighted control, Jacob’s focus was on mobility.

## The 1961 discussion: nonmutual understanding

McClintock’s controlling elements, as the name suggests, highlight the controlling effects rather than mobility. No wonder she aligned her work with the regulator-operator system instead of episomes, which sparked discussions in 1961. Regarding the scientific community’s reception of McClintock’s work, Nathaniel Comfort points out that geneticists already accepted that some elements could move but did not appreciate the control systems. Upon reading Jacob and Monod’s 1960 paper, McClintock “‘went through the ceiling’ with delight” (Comfort, 2001b, p. 207), recognizing a great opportunity to advance her colleagues’ understanding of maize control systems. She immediately convened a staff meeting at Cold Spring Harbor titled “Recent discovery of controlling elements in bacteria” in November 1960.^6^ According to the notes, McClintock introduced Jacob and Monod’s terminologies including “Structural Gene,” “Operator,” “Regulator,” and the “O-R System” (shorthand for regulator-operator system) and applied them to her own research. She also detailed how Jacob and Monod deduced the system from mutant behaviors.^7^ Shortly thereafter, McClintock published a paper in 1961 titled “Some Parallels Between Gene Control Systems in Maize and in Bacteria.” Jacob and Monod “were glad of her prompt support” (Judson, 1979, p. 461). They presented at the 1961 Cold Spring Harbor Symposium and cited “Parallels” even before its formal publication. However, published papers reveal that though McClintock comprehended Jacob and Monod’s research, the understanding was not mutual–they failed to grasp maize control systems, while reinforcing the perception that McClintock’s contributions were limited to mobile elements.

In “Parallels,” McClintock manifested a solid understanding of the regulator-operator system. The paper began by introducing Jacob and Monod’s concepts of regulator gene, operator gene, structural gene, and the repressor. McClintock stated: “it is apparent that a relationship may exist between the bacterial and the maize control systems” (1987, p. 407), and outlined three reasons: First, the systems in both maize and bacteria are comprised of two controlling elements: one controlling element (*Ac*, *Spm* element, regulator) controls the other controlling element (*Ds*, a mutable locus, operator), which further controls other chromosomal segments (genetic elements, structural genes). Second, McClintock emphasized the specific correspondence between the two controlling elements: “In maize, as in bacteria, each ‘operator-regulator’ system is quite specific: an ‘operator’ element will respond only to the particular ‘regulator’ element of its own system” (pp. 407–8). *Ac*, *Ds*, *Spm*, regulator, and operator refer to elements with a particular controlling effect. There are multiple *Spm* elements in maize and multiple regulator genes in bacteria, while each *Spm* element/ regulator controls a specific mutable locus/ operator. Third, McClintock observed that controlling elements in maize underwent mutations similar to those observed in the bacteria, and highlighted the *Spm* system as particularly illustrative of this resemblance. Mutable loci such as a_1_^m−1^ could mutate and lead to heritable phenotypical changes, which mirrors the mutation of the operator. McClintock’s discovery of mutants of the *Spm* element further supports this parallel: the *Spm*^*w*^ mutant exhibits weakened control and results in smaller areas of anthocyanin blotches, echoing the mutations of the regulator gene *i*^+^ to *i*^*−*^ or *i*^*s*^. Moreover, mutations in both the *Spm* system and the regulator-operator system are reversible, underscoring their similarity. Building on these parallels, McClintock further proposed that the *Spm* element could produce a repressor substance to “turn off” gene action at the mutable locus, akin to the regulator producing a repressor to suppress the operator.

McClintock also addressed Jacob’s episomes argument. She acknowledged that maize controlling elements sometimes transpose, but this behavior does not always occur: “transposition does not necessarily characterize the behavior of a controlling element. An element previously exhibiting transposition may become fixed in location” (p. 408). She reiterated that transposition, while enabling the recognition of controlling elements, does not serve as the fundamental characteristic. McClintock highlighted the existence of a specific type of *Spm* system, the class II state of the *Spm* system, which comprises non-transposing elements: “A class II state, with its operator element fixed in position and an *Spm* element that also is fixed in position, gives rise to a system of control of gene action in maize that simulates in its mode of operation some of the described systems in bacteria” (p. 418). Transposability is secondary, and the fundamental parallel lies in the controlling effects.

François Jacob and Jacques Monod presented at the Cold Spring Harbor Symposium on Quantitative Biology, “Cellular Regulatory Mechanism,” held from the 4th through the 12th of June in 1961. The symposium centered their presentations: “On the Regulation of Gene Activity” and “General Conclusions: Teleonomic Mechanisms in Cellular Metabolism, Growth, and Differentiation,” which were later revised and published (Jacob & Monod, 1961b; Monod & Jacob, 1961). McClintock was then working at the Cold Spring Harbor Laboratory; though not a presenter, she was listed among the attendees. Jacob and Monod acknowledged McClintock by citing her works “Parallels” and “Controlling Elements and the Gene (1956)” in the published versions of their presentations. Despite their acknowledgment, the papers show that they did not fully grasp McClintock’s research.

In “Regulation,” Jacob and Monod introduced more regulator-operator systems in *E. coli*,^8^ mentioning McClintock in the conclusion: “[evidence] for the existence of genetic systems of control has already been reported in maize” (Jacob & Monod, 1961b, p. 207). They cited “Parallels” and acknowledged that the control systems are two-element and specific, and “may, in some respect, be compared with the regulatory gene-operator system of bacteria.” Nevertheless, they emphasized mobility over control:

An important difference between the two systems is that, whereas in bacteria both regulators and operators appear as permanent, non-dispensable constituents of the genome, located at precise, constant loci of the genetic map…, the controlling elements of maize, at least some of them, seem to be dispensable and able to move from one chromosomal location to another. In this respect, the maize controlling elements behave like certain particular types of genetic elements in bacteria, called *episomes.* (pp. 207–208)

Jacob and Monod refuted McClintock’s parallels, insisting mobility as “an important difference,” and the paper continued to expand on episomes. In contrast to McClintock’s thorough elaboration of the regulator-operator system, this brief excerpt does not differentiate between the *Ac-Ds* system, where both *Ac* and *Ds* can move, and the *Spm* system, where only the *Spm* element can move, let alone the class II state of the *Spm* system with both controlling elements fixed. It is unclear whether they intentionally homogenized different systems to underscore mobility, or they failed to grasp the distinctions between control systems in maize.

“General Conclusions” suggests a lack of comprehension. Notably, Jacob and Monod co-wrote the first draft, and Monod spent months rewriting the manuscript before publishing.^9^ The paper is divided into two sections: “regulatory mechanisms” specifically in bacteria and “regulation and differentiation in higher organisms.” McClintock is only addressed in the first section, after reintroducing the regulator-operator system:

Long before regulator genes and operator were recognized in bacteria, the extensive and penetrating work of McClintock (1956) had revealed the existence, in maize, of two classes of genetic “controlling elements” whose specific mutual relationships are closely comparable with those of the regulator and operator: the “Activator” of McClintock appears to work as a transmitter of signals, presumably cytoplasmic since they act both in *cis* and in *trans*. By contrast the specific *receiver* of these signals only act in *cis* upon genes directly linked to it. (pp. 394-395)

McClintock’s 1956 paper is acknowledged as “extensive and penetrating”—indeed, she was the first to propose the concept of genetic control by distinguishing controlling elements from gene elements. However, even after several revisions, this quote remains confusing: It distinguishes the two classes of controlling elements not by their targets (the first class controls the second class; the second class controls gene elements), but by their action sites: the first acts on multiple chromosomes–“in *cis* and in *trans*;” the second solely acts on the chromosome where it resides–“in *cis*.” Nevertheless, action sites cannot serve as the criterion to distinguish controlling elements: in the *Ac-Ds* system, both *Ac* and *Ds* can transpose and act on multiple chromosomes; in the *Spm* system, the *Spm* element only controls a specific fixed locus, meaning neither the *Spm* element nor the mutable locus act in *trans*. The quote also refers to the first class as the “Activator,” but McClintock used the term “Activator” specifically to denote the *Ac* element, which activates the *Ds* element. It was not meant as a general reference to all controlling elements that regulate other controlling elements. This distinction is significant because the controlling effect is not always activation – in the *Ac-Ds* system, *Ac* activates *Ds*, but in the *Spm*-a_1_^m−1^ system, *Spm* suppresses or mutates a_1_^m−1^. McClintock distinguished between the two kinds of control systems in her 1956 paper, which this quote cites. Moreover, the quote claims that the Activator is “presumably cytoplasmic,” while McClintock never associated *Ac*, or any controlling elements, with the cytoplasm. Instead, she emphasized them as part of the genome as the plants follow Mendelian inheritance in cross experiments. It’s crucial to note that maize, a eukaryotic organism unlike the prokaryote *E. coli*, contains a nucleus. It is one thing for elements to transpose from one chromosome to another *within* the nucleus, but quite another to travel *outside* the nucleus into cytoplasm.

The acknowledgement reveals that Jacob and Monod did not understand McClintock’s control systems, and they also overlooked her insights into organism development as the second section, “regulation and differentiation in higher organisms,” ignores McClintock. Higher organisms undergo differentiation, where multiple cells with the same hereditary material develop into various forms. McClintock proposed in “Parallels”: “Specific types of mutation occurring at given times during the development and produced by a control system, such as the *Spm* system, may effect tissue differentiation along certain paths” (1987, p. 418). Relating to her 1951 paper and the Nobel lecture, McClintock kept a lifelong interest in differentiation and organism development, highlighting the pivotal role of control systems in these processes. While acknowledging that bacterial control mechanisms may not directly apply to higher organisms, Monod and Jacob insisted: “[it] may be in the interpretation and analysis of differentiation that the new concepts derived from the study of microorganisms will prove of the greatest value” and dedicated the second section to six hypothetical microbial models without mentioning McClintock’s work (p. 397). To conclude the 1961 discussion, while McClintock manifested a solid understanding of the regulator-operator system and even Jacob and Monod’s research process, maize control systems did not receive equal appreciation from the two bacterial geneticists. The understanding was not mutual.

## Aftermath: McClintock never gave up

The 1961 discussion marked McClintock’s attempt to control the legacy of her research—to gain peer recognition for maize control systems. The attendees at the 1951 Cold Spring Harbor Symposium remembered that “it was the control theory, not transposition per se, that they could not understand” (Comfort, 1999, p. 146). The scientific community were convinced that some elements could transpose but viewed them as random and meaningless events. A paradigm shift was needed to illuminate control. When McClintock read Jacob and Monod’s 1960 paper about the regulator-operator system, she was delighted by the parallels between their work and hers—control. Recalling the staff meeting at Cold Spring Harbor where she first presented the parallels, McClintock stated: “They were very excited; some people changed their line of work on the spot;”^10^ “For several people in the audience it changed completely their research–two people in bacterial and phage at the time [changed] their attitude immediately, and they saw immediately a new approach and changed with that.”^11^ At this moment, the operon appeared as an unparalleled opportunity to illuminate maize control systems.

Jacob and Monod were glad of McClintock’s prompt support, but they failed to comprehend the maize systems. This lack of comprehension led Jacob and Monod to reject McClintock’s parallels and reinforce mobility as the essential characteristic of controlling elements. Their response greatly influenced the scientific community: “In the 1950s geneticists held McClintock’s controller argument judiciously at arm’s length in the 1950s. In the 1960s they rejected it. The reason was the operon” (Comfort, 1999, p. 151). The concept of genetic control was accepted, and the operon soon won the 1965 Nobel Prize in Physiology or Medicine. However, instead of illuminating the maize systems as McClintock had anticipated, the operon eclipsed her work. This resonated with a linguistic shift from “controlling elements” to “transposable elements” in the 1960s (Comfort, 1999, p. 137), burying traces of the McClintock myth: Transposable elements, initially rejected in the 1950s, resurfaced in bacteria in the mid-1960s as “Insertion Sequences (IS).” Meanwhile, medical research uncovered the connection between transposable elements and the transformation of normal cells into tumor cells.^12^ McClintock’s initial discovery earned her an honorary Nobel in 1983, and she was celebrated as the fortunate Mendel who witnessed her own rediscovery. The Nobel Prizes in 1965 and 1983 aligned with Jacob and Monod’s interpretation of McClintock’s work, overshadowing her contribution to the concept of genetic control and her pioneering research on control systems.

Jacob and Monod rarely mentioned McClintock after 1961. Though Monod first met McClintock at the California Institute of Technology around 1936,^13^ they did not seem to correspond.^14^ Nevertheless, all three were interviewed by Horace Judson in the 70s for his book *The Eighth Day of Creation* which explores the history of biology in the twentieth century. A brief note from Monod’s interview in 1970 was the only evidence of Jacob and Monod mentioning McClintock: “1961: regulation of gene expression was at the [p]oint of transcription (see Miss McClintock).”^15^ On the other hand, McClintock talked about Monod: “In’61 Monod had to hammer [control] at people or they wouldn’t see.”^16^ Based on the interviews, Judson’s unpublished manuscript did not mention McClintock in the operon chapter, while the next chapter briefly addresses the 1961 discussion:

McClintock realized that the controls she had been attempted to bring out in maize were similar to the scheme they put forward for E. coli. She pointed out the parallels to them, and wrote a paper, which appeared at the end of the summer and was not ignored. Jacob and Monod had not cited McClintock’s work, this was an unhappy oversight symptomatic of their narrow focus on bacteria, Monod once told me. They were glad of her prompt support. In the printed version of Monod’s concluding summary for the Cold Spring Harbor meeting, which he and Jacob wrote together, they said…^17^

It continues with Jacob and Monod’s acknowledgement in “General Conclusion.” According to Judson, Jacob and Monod did not cite McClintock in the three operon papers, “an unhappy oversight,” but did not ignore her “Parallels” and made up for their oversight by acknowledging McClintock at the 1961 symposium. This manuscript was sent to McClintock in 1978. She heavily edited it and hand-wrote on the margin: “They did not understand the technical aspect of maize genetics. It was not an oversight but rather a lack of comprehension of the meaning of the data”(the double underline is original). It was not an oversight that could be compensated by a brief acknowledgement, but a lack of comprehension which continued to disturb McClintock even after seventeen years, and she telephoned Judson a few times “to voice alarm at errors” on this page. The published version of *The Eighth Day of Creation* in Judson 1979 modified the account: “…Jacob and Monod had not cited McClintock’s work–an unhappy oversight, Monod once told me. (“They did not understand the technical aspects of maize genetics,” McClintock said.) They were glad of her prompt support…” (p. 461). McClintock’s alarm was placed in brackets, maize control systems were eclipsed, while the myth about rediscovering mobile elements was perpetuated by the 1983 Nobel Prize.

Jacob and Monod quickly moved on from the 1961 discussion, while McClintock never gave up the parallels with the operon. She closely followed their work and appropriated the language of “regulator” and “operator” in her 1960s writings.^18^ McClintock was also committed to passing on her ideas to students. As an independent researcher at Cold Spring Harbor Laboratory, she embarked on teaching tours at various universities. In December 1961, McClintock conducted a seminar at Columbia University titled “Some Aspects of Systems of Control of Gene Action in Maize,” devoting the first section, “Review of Controll [*sic*] systems in Bacteria and Phage,” to the regulator-operator system. The third section, “The Maize Cases: Particular cases,” applied “O” and “R” (Jacob & Monod’s abbreviations for operator and regulator) to maize, featuring a subsection titled “Like the o i at the Lac locus,” addressing the parallel.^19^ Three months later, McClintock taught a seminar at Yale, “Systems Controlling Gene Action in Maize and Bacteria.” The third section, “The relation between control systems in bacteria and those in maize,” highlighted the parallels between the operon and the *Spm* system. McClintock also lectured on genetic systems in bacteria and eukaryotic organisms at Michigan in 1969.^20^ She likely presented the parallels on multiple occasions to influence the young generation.

Though McClintock stayed committed to the parallels between maize and bacterial systems, she grew critical of geneticists’ reliance on the operon to explain all control mechanisms in the late 1960s. The maize geneticist Oliver Nelson wrote to McClintock in 1968: “I’m not, however, sold on the similarities between controlling elements and either an episome model or an operon model. The dissimilarities appears [*sic*] sufficient that I’d like to reserve judgment suspecting that neither are applicable.”^21^ Neither episomes nor operon, did Nelson think that control systems in maize can parallel with. McClintock responded:

I agree with your comments...I have never thought that the episome model could be applied directly…I do not believe that controlling elements in maize may be homologized with models based on the current dogma of the operon. Nevertheless, in the examined cases in maize, it is quite evident that a component is present at the locus of a gene that controls the action of this gene and that alters this action in a distinctive manner in response to a second element. The specificity of a system, however, is analogous to the specificity of the operator and regulator system in bacteria.^22^

McClintock agreed with Nelson that episomes had no connection to control systems and maintained that maize systems were “analogous to” the operon, while cautioning against homogenizing maize control systems with the operon. She was criticizing the phenomenon where the operon not only eclipsed maize control systems, but created a barrier for the scientific community to appreciate control systems in prokaryotic organisms, as she reiterated in a 1979 interview:

That to me is an extremely important situation – why the eucaryotics were just thrown to one side and this information they were getting in the bacteria was carried over as far as regulations were concerned. All of the regulation was just thrown right over into eucaryotics, and it wasn’t going to work there, and the people didn't recognize it and they tried very hard in the eucaryotes to make operons, and all of that situation, work. They hadn’t realised that the eucaryotes are made up of a lot of cells and no two cells in different parts of the organism can be doing the same thing. They must be doing different things. Therefore there must be controls that are very different from what you get in the bacteria.^23^

Geneticists canonized and forced the operon on eucaryotes, which disturbed McClintock. During the 1983 roundtable discussion with Nobel Laureates in the Sciences, the host asked: “Do you think scientists sometimes tend to fall in love with a particular theory?” McClintock smiled and replied:

In a way, yes… The reason I say yes, is that I remember when gene regulation was first announced by Jacob and Monod, people were so sure that it was all it was to it that they said: “if you know bacteria, you can explain an elephant.” That didn’t come out right in the long run. But that was accepted by many people. I think we have to go through phases, when you clear up one generalization, and you find you have to go to another, and you see something shows what the other is going to be. But it never, never, never ends.^24^

It was Monod who made the famous bacteria-elephant comment. Though never backing off the parallels, McClintock was deeply disturbed by geneticists’ canonization of the operon.

Despite her persistent efforts through papers, lectures, and interviews, McClintock sometimes felt discouraged by her limited impact on the scientific community. Ernst Caspiri, one of her long-term correspondents, encouraged her by referring to Theodosius Dobzhansky, who was blessed with a late harvest for “saying and writing the same thing over and over again for the last 20 years.”^25^ McClintock replied in a desperate mood: “Again, I should add that without students to confirm and independently report their studies, I could not overcome the misconceptions and misinterpretations of my studies that are evident in the publications of others. Dobzhansky had students and post-docs to support his evidence and conclusions. I had none; I was in a hopeless position.”^26^ After years of explaining her work, misconceptions and misinterpretations persisted. In 1978, McClintock recalled the discouragement she felt in the 1961 discussion: “But [Jacob and Monod] didn’t understand mine, and I never gave another seminar, and haven’t. Since 1960. On that aspect of my work.”^27^

Jacob and Monod’s lack of comprehension significantly influenced the reception of McClintock’s work. It is crucial to reflect on why they failed to understand maize control systems, especially given the shared logic of genetic control. Historiography offers three possible explanations: First, McClintock preferred “factor” and “chromosomal locus” over the more common term “gene.” She avoided using “gene” during her presentation at the 1951 Cold Spring Harbor Symposia (Comfort, 1995, p. 1164). She distinguished controlling elements from gene elements and used “operator element” instead of “operator gene” in “Parallels,” indicating a reluctance to classify controlling elements as genes. This might have perplexed molecular geneticists who emphasized clear classifications of genes, RNAs, and proteins based on chemical components. Second, McClintock took a cytogenetic approach, relying on evidence including kernel phenotypes, pictures of chromosomes, and maps of chromosomal loci. Comfort (1999, 2002) argues that none of these provided direct proof of the existence of control systems, leading to geneticists’ skepticism. Unlike the *Ds* element, whose mobility could be observed through broken chromosomes, control systems were deduced rather than directly observed. Only until the 1980s did molecular biologists, with Nina Federoff as a leading figure, begin to visualize controlling elements through sequencing.^28^ On this point, I suggest that Jacob and Monod also deduced, rather than observed, the regulator-operator system from biochemical data. Their evidence was *E. coli*’s “phenotype”–whether it produces proteins. They did not *observe* any mutants, the operator, or the regulator, as gene sequencing only developed in the 1970s. On this point, Evelyn Fox Keller (1983a) and Carla Keirns (1999) provide a more nuanced interpretation: McClintock was speaking a different language. Photos of kernels and chromosomes differed from Jacob and Monod’s charts of enzyme density, and McClintock never drew cartoon-like diagrams to illustrate control systems. Nonetheless, McClintock’s language was not entirely incomprehensible, as some molecular biologists, such as Kenneth Paigen, were able to understand her work, as will be demonstrated in the following section. Third, McClintock used a less favored model organism, maize. Melvin Green, a *Drosophila* geneticist and a friend of McClintock, recalled that until the 1950s, maize and *Drosophila* served as prominent model organisms in genetics. However, with the rise of biochemical techniques, microbes became the new focus. In 1953, a phage geneticist even declared to Green that “[*Drosophila* is] a dead organism!” and “The action is with the microbes.”^29^ Given Jacob and Monod’s pioneering work on bacteria and phages, they might have felt reluctant to invest time in understanding maize. The power dynamics and disciplinary disparities between cytogenetics and molecular biology in the 1960s offer insight into why McClintock carefully reviewed Jacob and Monod’s papers, whereas they failed to comprehend hers. I propose another factor, gender.

## Reflection: three-fold Matilda effect

McClintock was not only an outstanding geneticist, but also a prominent women scientist. Two biographies, Evelyn Fox Keller’s *A Feeling for the Organism* (1983a) and Nathaniel Comfort’s *The Tangled Field* (2001b), interpret gender differently. As a woman scientist herself (Creager, 2023), Keller wondered why the scientific community favored certain explanations over others. For example, regarding slime mold aggregation, she questioned why biologists were fixated on the idea that certain cells initiated the process, rather than considering the possibility that all cells were homogeneous and simply aggregated (Keller & Segel, 1970; Keller, 1983b). During in-person interviews, Keller perceived a similar issue with McClintock. She suggests that geneticists preferred the notion of a stable genome rather than considering that some elements could move, leading Jacob and Monod to overlook the concept of transposability (1983a, p. 10). Keller posits that McClintock’s research embodies gender-neutral science, characterized by a feeling for the organism, which led to her marginalization by the scientific community: “Above all, *A Feeling for the Organism* is the story of one woman’s conception of science that gradually—although not irrevocably–isolated her from the evolving discourse of mainstream research” (p. xv). On the other hand, Comfort carefully examined McClintock’s papers and research notes and claims: “Keller took McClintock at face value” (2001b, p. 5). He shows that it was the control systems, rather than transposability, that geneticists rejected. Comfort contends that gender hardly contributed to this rejection: “Nothing in McClintock’s story denies the prejudice, discrimination, and marginalization experienced by other women, either in or out of science. But she is a poor symbol for those hardships,” and “Nor does her story support the idea of a nonmasculine science” (p. 10). According to Comfort, McClintock’s control systems lacked sufficient evidence and thus failed to persuade Jacob, Monod, and other geneticists in the field of science–one plausible interpretation of the book title, “the tangled field.”

The 1961 discussion showcases McClintock’s emphasis on control rather than mobility, while I posit that Margaret Rossiter’s concept of the Matilda effect offers insight into the dynamics of McClintock’s career, particularly evident in the 1961 discussion. Women scientists in the U.S. faced significant challenges from the 1940s to the 1960s: Though World War II provided opportunities for women in scientific and technical projects, the post-war period witnessed a regression, with even women’s colleges seeking to diminish their presence. As Rossiter notes, the few survivors “were at best invisible and at worst an embarrassment” (1995, p. xvi). Named after Matilda Joslyn Gage (1826–1898), who offered a feminist interpretation of the Bible, Rossiter defines the Matilda effect: “the sexist nature of much of the women’s systematic under-recognition should be acknowledged, noted and even highlighted in the sociology of knowledge or science” (1993, p. 337). At first glance, McClintock seemed an exception to the Matilda Effect – she achieved remarkable success and received widespread recognition. She even enjoyed the “odd” luck of being the sole female student at the agriculture college at Cornell.^30^ Before the 1983 Nobel Prize, McClintock served as the president of the Genetics Society of America in 1945 and had harvested numerous awards, including the National Medal of Science in 1970, the Lewis S. Rosenstiel Award in 1978, and Thomas Hunt Morgan Medal and Albert Lasker Basic Medical Research Award in 1981. As for the 1961 discussion, no archival evidence suggests that Jacob or Monod made misogynous comments about McClintock–they rarely mentioned her in letters and interviews. Nevertheless, I argue that the Matilda effect influenced McClintock; it is a reminder that even highly accomplished women scientists like McClintock faced gender bias and struggled to have their contributions properly acknowledged and understood. I delineate the sexist nature of under-recognition into three dimensions: social, intellectual, and epistemological. Regarding the last, while Keller misconstrued McClintock’s research focus, the concept of gender-neutral science offers insight into the 1961 discussion.

In the social dimension, McClintock recalled the hardships she faced during her college years and the sex discrimination she encountered in the job market: “You really were stigmatizing yourself a bit by being a spinster and a career woman, especially in science.”^31^ There was an anti-female tradition at Cornell when McClintock was a graduate student. She served as an assistant to a male professor who worked on maize chromosomes in her second year. When she found a better way to distinguish the chromosomes, the professor got furious; when she offered to edit a dreadful manuscript sent to *Genetics*, he wrote a three-page letter calling her “everything under the sun.”^32^ Regarding the job market, McClintock left Cornell University in 1931 because the only available position was an embarrassing instructor, as she was a woman.^33^ She eventually landed at the Cold Spring Harbor Laboratory in 1941, working for the Carnegie Institute of Washington, a non-profit institution offering relatively low salaries. Rossiter notes that during the 1950s and 1960s, only about 1 percent of American scientists and engineers worked at non-profit institutions, while these institutes were often the primary option for women scientists who would otherwise have been unemployed Rossiter (1995 p. 235). The Matilda effect endures even after women scientists have secured prestigious positions. McClintock recalled an insulting offer she received around 1948 when she was already established and had served as the president of the Genetics Society of America. A university department “wanted to build up the prestige” and offered McClintock the position of “the only assistant professor” with a salary even one third lower than what she received from the Carnegie Institute of Washington. The offer was a serious attempt, which disturbed McClintock: “I felt that [women], in any kind of a profession, had been much neglected, except in the entertainment world… these people couldn’t recognize what they were doing. That’s what I was disturbed about, not about me personally because it didn’t make any difference—I wasn’t being insulted. Women were being insulted.”^34^

Rossiter defines the Matilda effect as the reverse of the Matthew effect—the cumulative advantage especially of intellectual property, notably peer recognition via citation (Merton, 1988). Jacob and Monod did not cite McClintock in their three operon papers–an “unhappy oversight”–and refuted her parallels. Their under-recognition influenced the scientific community. While it is difficult to speculate on the potential impact Jacob and Monod could have made, I suggest that the 1961 discussion bears similarities to “a coincidence” that McClintock frequently brought up during interviews.^35^ When serving as an instructor at Cornell in the 1920s, she cross-bred maize and studied the chromosomes, but her colleagues were unable to grasp her research and thought that she was “a little mad.” Cornell nearly dropped McClintock, and she lost her only collaborator. It wasn’t until Marcus Rhoades arrived in 1928 for his Ph.D. in genetics that McClintock found support. Fascinated by her work, Rhoades joined her research and explained its significance to others, and McClintock was “taken back into the fold as a consequence.” McClintock’s colleagues accepted the explanations given by Rhodes, a male Ph.D. student. Unlike the 1961 discussion, Rhodes understood McClintock’s work, but it took more than good fortune for McClintock to be understood, as she recalled: “But it was not that he wanted to join me in this problem that I was working on. He wanted to get into the area of cytogenetics and here was an opportunity; he saw the opportunity and has stayed as a cytogeneticist with maize ever since.”

As established bacterial geneticists, Jacob and Monod appreciated McClintock’s support but not her research, though other less prominent bacterial geneticists did.^36^ One molecular biologist, Kenneth Paigen, reached out to McClintock in 1965 to discuss the similarities between the *Spm* system and some reversible mutations in *E. coli*: “I was very intrigued by a sentence on page 594 which indicates that Spm effects phenotypic expression in progeny kernels that do not receive Spm…I would be very curious to know whether you think there might be any conceptual relationship between host control (or host modification) and the Spm effect.”^37^ Paigen’s detailed question suggests that McClintock’s papers were not incomprehensible to molecular biologists. McClintock soon responded with enthusiasm: “Change in control of gene action, induced by Spm but subject to reversal some cell generations later in the absence of Spm, is suggestive of a parallel. Right now, the concept of the parallel is fuzzy in my mind but if we discuss it, possibly something may crystalize. Let’s talk about it!”^38^ They probably had a fruitful discussion that summer at Cold Spring Harbor and stayed in touch afterward. While Paigen tried to vouch for McClintock, his attempt was not as successful as Rhodes’s in the 1920s. In 1976, Paigen prepared a Nobel nomination for McClintock, which included a four-page description of her contributions:

In her discovery of genetic regulatory elements, or as she termed them “controlling elements”, Dr. McClintock initiated a new era in genetic research. In addition, her early recognition that genetic regulatory systems use two fundamentally different classes of regulatory genes was crucial in laying the groundwork for later investigations in microbial systems. Unable to use molecular approaches that were still years away, she turned to the use of sophisticated genetic mapping techniques to distinguish between elements able to initiate changes in gene expression at distant sites and elements required to mediate these changes in adjacent structural genes, that is to say regulatory elements that generate a signal acting over a distance and operator-like elements that receive the signal and thereby influence the expression of an adjacent structural gene.^39^

According to Paigen, McClintock’s concept of genetic control “initiated a new era in genetic research,” and her control systems serve as the precursor of the operon. He sent the materials to James D. Waston asking for a supporting letter.^40^ Waston replied four months later and declined, offering: “When George Klein is here in early September, I’ll ask him whether it would help to mount a real lobbying effort to Barbara.”^41^ It is unclear whether Paigen successfully submitted the nomination. Even if he did, it ultimately proved unsuccessful.

McClintock’s control systems did not receive widespread peer recognition, but was such under-recognition *due to* her identity as a woman? Nathaniel Comfort argues no: “Though McClintock, like nearly all women at the time, was subject to entrenched, cultural gender discrimination, she seems to have been treated ‘like a man’ much more than women. Science is more gender-blind at the top than in the middle ranks” (Comfort 1999, p. 153). Nonetheless, science is not immune from cultural influences such as gender. Drawing from the 1961 discussion, I argue that the Matilda effect operated in an epistemological dimension as well–Keller’s concept of gender-neutral science helps explain why Jacob and Monod failed to appreciate maize control systems. Keller defines: “My vision of a gender-free science is not a juxtaposition or complementary of male and female perspectives, nor is it the substitution of one form of parochiality for another. Rather, it is premised on a transformation of the very categories of male and female, and correspondingly, of mind and nature” (1985, p. 178). Gender-neutral science transcends the binary of female and male and can be achieved by both women and men scientists, embodied in a different understanding of nature. Keller is critical of the concept of “feminist science,” highlighting the political danger inherent in the term: “Feminist science” implies sexual determinism, suggesting that only women scientists can pursue feminist science, and that they tend to approach science differently than their male counterparts. This implication harms women scientists because a different science almost always means a *lesser *science (Keller, 2014). Moreover, “feminist science” is historically inaccurate as McClintock rejected identification as a feminist (Keller, 1987). Indeed, McClintock perceived herself as a maverick, different from most men and women. Keller coined the term “gender-neutral science” to convey the transcendence of masculinity and femininity: “it is the reclamation, from within science, of science as a human instead of a masculine project, and the renunciation of the division of emotional and intellectual labor that maintains science as a male preserve…in order to be more objective” (1985, p. 178).

McClintock’s research embodies gender-neutral science, a perspective that becomes clearer when contrasted with Jacob and Monod’s views of nature. While their scientific papers share common ground—all argue for genetic control, propose similar systems, and employ terms such as regulation and information, their disparities come to light in interviews, Nobel lectures, and philosophical writings. McClintock’s perspective differs from Jacob and Monod’s in two interrelated ways: she recognized organisms’ diversity and agency, thereby acknowledging the inherent limitations of human understanding. Regarding the former point, McClintock described her research approach as “the feeling for the organism”:

I've got to know every one, and you get so you do get a feel for any individual plant, because no two are exactly alike – some of the hybrid stuff you see growing in the fields, an awful lot of them look exactly alike, almost alike, they're very much alike. So homogeneous. But the plants I grow – every one is distinct. No two plants are exactly alike. They're all different, and as a consequence, you have to know that difference. That's what I mean: you have to know it in order to make the best use of the plant.^42^

Maize plants may seem uniform, but a discerning geneticist can feel the difference. McClintock recognized various phenotypes, differentiated kernels with spots from those with blotches, and perceived diverse control systems such as the *Ac-Ds* and the *Spm*. Marveling at the uniqueness of each individual organism, she expressed awe: “Even the smallest organism, I think, even bacteria, *very* intelligent, very smart. I don’t yet understand the degree of smartness of these systems we call living.”^43^ Her profound respect for organisms extended to bacteria, which she viewed as intelligent and beyond complete comprehension. Control systems in maize demonstrate the plant’s intelligence–its genome can adapt to environmental changes, as discussed in her Nobel lecture “The Significance of Responses of the Genome to Challenge.” The emphasis on plant autonomy explains why McClintock never drew parallels between controlling elements and episomes from outside the organism. In her interview with Horace Judson, McClintock highlighted the agency of maize: “The material just tells you where to go. The maize cases were so spectacular that I just had to follow them.”^44^

On the other hand, Jacob remarked: “For the present time I am just playing with cells.”^45^ The molecular biologist takes dominance. Molecular biology takes a reductionist approach (Keller, 1995): Higher organisms are reduced to single-cell organisms such as *E. coli*, further to particles such as DNA, RNA, and proteins, ultimately comprising homogenous molecules and atoms. This perspective suggests a preference for homogeneity over heterogeneity. Regarding the operon, Jacob and Monod inferred the system from mutant behaviors, while viewing these mutants as instances of failed self-regulation. In contrast to maize’s diverse control systems, the *normal E. coli* is characterized by the presence of a *standard* operon. After 1961, Jacob investigated the chemical nature of the repressor rather than explored control mechanisms beyond the operon. The repressor was identified as a protein that responds to both the inducer–lactose in the lactose system–and the operator gene. Meanwhile, Monod delved into the allosteric effects–how proteins alter their surface molecules in response to different substances.

Based on the reductionist approach, molecular biology implies a potential for understanding higher organisms through bacteria. While Jacob remained cautious on this front, likely due to his shift towards using mice as model organisms in the mid-60s (Méthot, 2023), Monod displayed confidence: “The secret of life? This is in large part known, in principle. For this to be synthesized, in my opinion there is no further principle that would need to be discovered.”^46^ He famously commented “anything found to be true of *E. coli* must also be true of Elephants” first in 1954 (Friedmann, 2004) and reiterated it in “General Conclusions”(Monod & Jacob, 1961, p. 393), where he proposed six hypothetical bacterial models to explain higher organisms’ differentiation and development. In his 1965 Nobel lecture, Monod asserted molecular biology’s ability to elucidate “the more complex biological phenomena: the development of higher organisms and their networks of functional coordinations” (1965, p. 207). This confidence extended to an explanation of human society in his monograph, *Le Hasard et la Nécessité* (*Chance and Necessity,* 1970), where he argued that while genes mutate by chance, genetic transcription and evolution necessitate the survival of the fittest individuals, species, and even human ideas. With such unwavering confidence in the abilities of bacterial geneticists, Monod may have felt little need to study maize, thereby rarely corresponding with McClintock.

McClintock’s reverence for organisms’ diversity and agency instilled in her a sense of humility. During the Nobel round table discussion, she frequently reiterated: “I don’t know.” Even bacteria, the smallest but not the simplest organism, remained unfathomable, leading her to criticize the canonization of the operon. For McClintock, science was not about asserting authority, but about enjoying doing research: “I’m not a great one to glorify science: finding these relationships has been great fun;” her open-mindedness led her to explore extrasensory perception (ESP) in 1952 “as a consequence of the arrogance of the biologists.”^47^ McClintock read books about esoteric traditions and frequently brought up this topic during interviews.^48^ Her view of science transcends the division of emotional and intellectual labor, embodying gender-neutral science as defined by Keller.

While both men and women can achieve gender-neutral science, Keller posits that women scientists are more inclined to embrace it due to the identity challenges in a scientific culture historically dominated by men: “In a science constructed around the naming of object (nature) as female and the parallel naming of subject (mind) as male, any scientist who happens to be a woman is confronted with an a priori contradiction in terms. This poses a critical problem of identity: any scientist who is not a man walks a path bounded on one side by inauthenticity and on the other by subversion” (Keller, 1985, p. 174). As a woman scientist, McClintock could not align herself with the reductionist approach or the assertion of omniscience – inauthenticity. She keenly felt the barrier between herself and male scientists: “I’m under this tension, and sometimes I feel the pressures from the men. There is a barrier that is erected between – I just do not have the ability to reach them, very often. There’s no question, it’s because I’m a woman.”^49^ She continued:

**McClintock**: …Well, when I was at Missouri, I was teaching graduate students, and there was a man named Herschal Roman…Very bright graduate student…We used to have our dinners together fairly often, and one time when we were having dinner, we were talking about the university in one way or another, and he said, “I can’t stand women professors, I just can’t stand them.” Well, I just let him go on a while, and then I said, “Herschal, to whom do you think you’re talking?” [laughs].

**Keller**: You did not tell me this story. And he said?

**McClintock**: Well, I don’t remember what he said, but I think it was an eye opener for him. You see, the point is this: when a person gets to know you well, they forget that you’re a woman…I know that everybody I’ve ever worked with, all the men I’ve worked with, have treated me completely as they would another man in the group.

The misogynist comment illuminated a bittersweet moment for McClintock. While she momentarily “passed” as just “another man in the group,” this passing showcased inauthenticity as she could not align with the comment. Therefore, she pointed this out, much like she did with the canonization of the operon. The incident was “an eye-opener” for the male student, indicating McClintock’s transcendence of gender stereotypes–an act of “subversion.” Faced with identity challenges, women scientists are compelled to develop gender-neutral science, whereas their male counterparts may stay in the comfort zone: “This is not to say that the male scientist cannot claim similar redefinition (certainly many have done so) but, by contrast to the woman scientist, his identity does not require it” (Keller, 1985, p. 175). It was through reclaiming science as a human endeavor that McClintock grasped Jacob and Monod’s operon, while they failed to appreciate maize control systems stemming from a feeling for the organism.

## Conclusion: to reverse the Matilda effect

The published papers from 1961 demonstrate that McClintock had valid reasons to draw parallels between maize control systems and the operon, a strategic move to garner peer recognition. However, Jacob and Monod’s lack of comprehension led to their rejection of the parallels, proposing that McClintock’s contribution was limited to mobile elements. The 1965 Nobel Prize credited genetic control to Jacob and Monod, while the 1983 prize reinforced McClintock’s association with mobile elements. The 1961 conversation serves as a reminder that the Matilda effect operates not only socially but also intellectually (peer under-recognition) and epistemologically (gender-neutral science). Keller posits that mobile elements embody gender-neutral science, which led to McClintock’s marginalization by the scientific community, while Nathaniel Comfort highlights McClintock’s focus on control systems instead of mobile elements. Integrating these perspectives, I suggest that control systems embody Keller’s concept of gender-neutral science. McClintock’s persistent frustration at not being understood stemmed from the under-recognition of control systems, not mobile elements. Consequently, McClintock’s story was not fulfilled by the 1983 Nobel Prize, nor did gender-neutral science receive peer recognition, but the Matilda effect endured—the under-recognition of control systems by the 1961 discussion and the two Nobel Prizes. Reflecting on her career, McClintock summarized in the introduction to her 1987 paper collection:

It was my intention in the summer of 1945 to attempt to find out what this regulatory event might be. It was not until fifteen years later that the regulation of gene action began to gain credibility due to the elegant experiments of Jacob and Monod that were carried out in bacteria…The integrated systems of transposable elements in maize, which I called “controlling elements” because of their distinctive modes of regulating the expression of genes, turned out to represent a quite different mode of gene regulation from that described by Jacob and Monod. Only now, more than forty years after the discovery of transposable elements, are we beginning to understand enough about the ways that they can affect genes to decipher some intriguing new aspects of gene control from their study (1987, p. xi).

McClintock became interested in genetic control as early as 1945. Once again, she recalled the excitement of reading about the operon and her unease with its canonization. Approaching the twilight of her career, she advocated for an understanding of the diverse controlling effects.

According to Rossiter, the Matilda effect operates both historically, as women scientists experience under-recognition, and historiographically, as they are often downplayed by historians and sociologists of science. In *The Eighth Day of Creation* Judson, (1979), McClintock is relegated to a footnote alongside Jacob and Monod, and Judson only briefly references the 1961 discussion. Her sole photo is a group picture of Monod in 1946 with the comment: “Monod was greatly tempted to become a proselyte to phage.” Nevertheless, McClintock has been the focus of two influential biographies–Keller (1983a) illuminates her view of science; Comfort (2001b) illustrates her research. McClintock has received great attention from historians and sociologists, appearing on the cover page of Rossiter’s *Women Scientists In America* (1995). The Judson 1995 edition of *The Eighth Day of Creation* include an epilogue highlighting McClintock’s discovery of mobile elements. McClintock’s story, though she never identified as a feminist and despite Keller’s critique of feminist science, sparked discussions about feminism and its impact on science. For instance, Longino (1987) posits that feminist science means politically choosing a different scientific model. Schiebinger (2000) highlights feminism’s influence on science, noting increased participation by women and the integration of women’s concerns into scientific research. Though McClintock suffered from the Matilda effect, her legacy provides strengths to reverse it.

## Footnotes

1 “The Nobel Prize in Physiology or Medicine 1983.” https://www.nobelprize.org/prizes/medicine/1983/summary/

2 “Nobel Lecture.” December 8, 1983; the italics reflect the speaker's tone. https://www.nobelprize.org/prizes/medicine/1983/mcclintock/lecture/

3 This paragraph is based on McClintock’s publications through the 50 s and the 60 s. McClintock first introduced the *Spm* element in “Mutations in Maize and Chromosomal Aberrations in Neurospora” (1954); “Genetic Systems Regulating Gene Expression during Development” (1967) includes photos that illustrate the relationship between phenotypes and the *Spm* system.

4 She hand-wrote the English translation, folder “Jacob, François and Jacques Mo. 1959,” Box 25, Series IV, Barbara McClintock Papers, American Philosophical Society (APS).

5 The 1961 paper also mentions the lysogenic system which contains a similar regulator gene. I focus on the lactose system as it is discussed in both the 1960 and 1961 papers.

6 File “Staff Meeting, November 28, 1960,” Folder “Cold Spring Harbor Lectures,” Box 14, Series III, Barbara McClintock Papers, APS.

7 For example, one subsection is titled “Discovery of the Operator: By mutations occurring to it:”.

8 For example, the system that produces the enzyme tryptophan-synthetase, and the bacterial immune system against phage.

9 Jacob’s interview with Horace Judson on May 5, 1978, p. 3. Folder “Jacob, François,” Box 1, Horace Freeland Judson Collection, APS.

10 Interview with Horace Judson on July 30, 1978. Folder “McClintock, Barbara,” Box 2, Horace Freeland Judson Collection, APS.

11 Interview with Evelyn Fox Keller on 1978.09.24, p. 59, Series VII, Barbara McClintock Papers, APS.

12 “The Nobel Prize in Physiology or Medicine 1983.” https://www.nobelprize.org/prizes/medicine/1983/press-release/

13 Interview on December 1, 1975, p. 3, Folder “Monod, Jacques” Box 2, Horace Freeland Judson Collection, APS.

14 Though Jacob and Monod corresponded frequently with other geneticists at Cold Spring Harbor such as James Watson and Sydney Brenner, there exists no correspondence between them and McClintock at Cold Spring Harbor and APS.

15 Interview in September 1970. Folder “Monod, Jacques” Box 2, Horace Freeland Judson Collection, APS.

16 Interview notes in June 1973. Folder “McClintock, Barbara,” Box 2, Horace Freeland Judson Collection, APS.

17 File “Jun. 1973, Cold Spring Harbor Laboratory Jul. 30, 1978, Cold Spring Harbor Laboratory n.d., her comments,” Folder “McClintock, Barbara,” Box 2, Horace Judson Papers, APS.

18 She read and took notes of Jacob and Monod’s presentation “Elements of Regulatory Circuits in Bacteria” for the UNESCO Symposium in September 1962. Box 25, Series IV, Barbara McClintock Papers, APS.

19 File “Some Aspects of Systems of control of Gene Action in Maize.” Box 18, Series III, Barbara McClintock Papers, APS.

20 File “Regulation of Gene Expression By Genetic Systems – Lecture.” Box 18, Series III, Barbara McClintock Papers, APS.

21 Nelson to McClintock on March 20, 1968, Folder “Nelson, O. E.,” Box 4, Series I, Barbara McClintock Papers, APS.

22 McClintock to Nelson on March 28, 1968, Ibid.

23 Interview with Evelyn Fox Keller on 1979.01.13, p. 61, Series VII, Barbara McClintock Papers, APS.

24 “Science and Man: A Round table discussion with the 1983 Nobel Laureates in the Sciences,” hosted by Bengt Feldreich and Jackie Klopp in 1983, 48min. PA_028_McClintock_Barbara, Cold Spring Harbor Archive (CSHL).

25 Caspiri to McClintock on April 14, 1973, Ernst W. Caspari Papers, APS.

26 McClintock to Caspiri on May 19, 1973, Ibid.

27 Interview with Keller on 1978.09.24, pp. 59–60, Series VII, Barbara McClintock Papers, APS.

28 Among the earliest publications on molecular analysis of controlling elements, see “Genetic and Molecular Analysis of the Spm-dependent a-m2 Alleles of the Maize a Locus” (Masson, Surosky, Kingsbury, & Fedoroff, 1987) as an example.

29 Interview with Melvin M. Green by Nathaniel Comfort, December 21, 1996. Transcript p. 6, Series VII, Barbara McClintock Papers, APS.

30 Interview with Keller on 1978.09.24, p. 27–32, Series VII, Barbara McClintock Papers, APS.

31 Ibid., p. 44.

32 Ibid., p. 41.

33 Interview with Keller on 1979.01.13, p. 16, Series VII, Barbara McClintock Papers, APS.

34 Ibid., p. 31–2; McClintock recalled this incident in other interviews, too.

35 For example, interview with Keller on 1978.09.24, pp. 38–40, Series VII, Barbara McClintock Papers, APS.

36 In addition to Paigen, James D. Wolf nominated McClintock for the Wolf Prize in Medicine in 1981. Folder “McClintock, Barbara,” Box 27, Series II, James D. Watson Collection, CSHL.

37 Paigen to McClintock on May 7, 1965, “Kenneth Paigen,” Box 5, Barbara McClintock Papers, APS.

38 McClintock to Paigen on May 24, 1965, “Kenneth Paigen,” Box 5, Series I, Barbara McClintock Papers, APS.

39 Folder “McClintock, Barbara,” Box 27, Series II, James D. Watson Collection, CSHL.

40 Paigen to Waston on 1976.04.21, ibid.

41 Waston to Paigen on 1976.08.09, ibid.

42 Interview with Keller on 1979.01.13, p. 54, Series VII, Barbara McClintock Papers, APS.

43 “Science and Man: A Round table discussion with the 1983 Nobel Laureates in the Sciences,” hosted by Bengt Feldreich and Jackie Klopp in 1983, 37min. PA_028_McClintock_Barbara, CSHL.

44 Interview on July 30, 1978. Folder “McClintock, Barbara,” Box 2, Horace Freeland Judson Collection, APS.

45 Interview on December 09, 1975. Folder “Jacob, François,” Box 1, Horace Freeland Judson Collection, APS.

46 Interview with Judson in September 1970, folder “Monod, Jacques,” Box 2, Horace Freeland Judson Collection, APS.

47 Interview with Judson in June 1973, Folder “McClintock, Barbara,” Box 2, Horace Freeland Judson Collection, APS.

48 Folder “book collection,” Barbara McClintock collection, Box 2, CSHL.

49 Interview with Keller on February 25, 1979, pp. 20–22, Series VII, Barbara McClintock Papers, APS.
